# Assessment and Diagnostic Classification Using DC:0-5 in Early Childhood Mental Health Clinics: The Protocol for the Developmental Psychiatry Diagnostic Challenges Study (DePsy)

**DOI:** 10.3390/children10111770

**Published:** 2023-10-31

**Authors:** Katja Bödeker, Laura M. Watrin-Avino, Annick Martin, Franziska Schlensog-Schuster, Marius Janssen, Lennart Friese, Maria Licata-Dandel, Volker Mall, Juliane Teich-Bělohradský, Yonca Izat, Christoph U. Correll, Eva Möhler, Frank W. Paulus

**Affiliations:** 1 Department of Child and Adolescent Psychiatry, Charité—Universitätsmedizin Berlin, Augustenburger Platz 1, 13353 Berlin, Germany; laura.watrin@charite.de (L.M.W.-A.); christoph.correll@charite.de (C.U.C.); 2 Department of Child and Adolescent Psychiatry, Psychotherapy and Psychosomatics, Leipzig University Medical Center, Liebigstrasse 20a, 04103 Leipzig, Germany; annick.martin@medizin.uni-leipzig.de (A.M.); franziska.schlensogschuster@upd.ch (F.S.-S.); 3 University Hospital of Child and Adolescent Psychiatry and Psychotherapy, University of Bern, Bolligenstrasse 111, 3000 Berne, Switzerland; 4 Department of Child and Adolescent Psychiatry, University of Münster, Schmeddingstraße 50, 48149 Münster, Germany; marius.janssen@ukmuenster.de (M.J.); lennart.friese@ukmuenster.de (L.F.); 5 kbo-Kinderzentrum Munich, Technical University of Munich, Heiglhofstraße 65, 81377 München, Germany; maria.licata-dandel@kbo.de (M.L.-D.); volker.mall@kbo.de (V.M.); 6 Department of Psychology, Charlotte-Fresenius-University, 80797 Munich, Germany; 7 Vivantes Clinic Friedrichshain, Child and Adolescent Psychiatry Berlin, Landsberger Allee 49, 10249 Berlin, Germany; juliane.teich@vivantes.de (J.T.-B.); yonca.izat@vivantes.de (Y.I.); 8 Department of Psychiatry, The Zucker Hillside Hospital, Northwell Health, Glen Oaks, NY 11004, USA; 9 Donald and Barbara Zucker School of Medicine at Hofstra/Northwell, Department of Psychiatry and Molecular Medicine, Hofstra University, Hempstead, NY 11549, USA; 10 The Feinstein Institutes for Medical Research, Center for Psychiatric Neuroscience, Northwell Health, New Hyde Park, NY 11030, USA; 11 German Center for Mental Health (DZPG), Partner Site Berlin, 10785 Berlin, Germany; 12 Department of Child and Adolescent Psychiatry, Saarland University Hospital, Kirrberger Straße 100, 66421 Homburg, Germany; eva.moehler@uks.eu (E.M.); frank.paulus@uks.eu (F.W.P.)

**Keywords:** DC:0-5, early childhood, child psychiatry

## Abstract

Mental health problems in early childhood are common, but there is a lack of psychiatric research on this age group. DC:0-5 is a multiaxial classification system for mental disorders in early childhood, providing a framework for standardizing clinical practice and research. However, research on the validity of DC:0-5 is scarce. The Developmental Psychiatry Diagnostic Challenges Study (DePsy) is a multi-site, prospective clinical study including six German early childhood mental health (ECMH) clinics. The main objective of the study is to contribute to the validation of Axis I and Axis II of DC:0-5. A second aim of the study is to describe the population of the participating clinics regarding diagnoses, family context, and treatment outcomes. Additionally, the impact of environmental risk factors, including parental Adverse Childhood Experiences (ACEs) and media use, on child psychopathology and caregiver–child relationships will be examined. Over two years, patients aged 0.0–5.9 years old will be enrolled in the study. Assessments include ICD-10 and DC:0-5 diagnoses, developmental tests, video-based observations of caregiver—child interactions, and questionnaires on child psychopathology, media use, parental stress, and treatment satisfaction. Study results will promote the standardization of assessment and treatment in ECMH clinics aiming to improve the development of patients and their families.

## 1. Introduction

Epidemiological studies indicate a frequency of mental disorders of 16–18% in preschool children (slightly more than half with more severe impairments) [1,2,3]. Given the high prevalence of mental health disorders in infants, toddlers, and preschool children, early childhood mental health (ECMH) clinics have become embedded within child psychiatric clinical care in many places. However, there is still a paucity of research on both the care services and the population, and a lack of standardization makes it difficult to compare results [4]. In order to optimize clinical care in ECMH, comprehensive studies on psychopathology, diagnoses, the demographic characteristics of families, and their access to support systems, are needed. Furthermore, clinically oriented research should focus on the implementation and effectiveness of the very specific interventions recommended in ECMH clinics.

Diagnostic classification systems provide a framework for the standardization of clinical care in ECMH. Correspondingly, the *Diagnostic Classification of Mental Health and Developmental Disorders of Infancy and Early Childhood*, abbreviated to DC:0-3, published in 1994 [5], and its revision DC:0-3R, published in 2005 [6], stimulated treatment and research on mental disorders in infancy and early childhood, as both systems provided a developmentally sensitive and explicitly relational classificatory framework. The most recent revision, DC:0-5, published in 2016 [7], diverges significantly from its predecessors. However, up to now, research on the DC:0-5 is scant.

In early childhood, there is a complex interplay between individual psychopathology, development, the child’s close relationships, and the environment. As reflected in the DC:0-5 multiaxial approach, all of these factors should be considered when assessing and treating preschool children. Understanding the psychopathology of the individual child in the context of close dyadic relationships with caregivers has been a vital part of infant psychiatry from its earliest days. However, the parent–child dyad does not stand on its own, and is subject to parental and environmental stressors, which in turn may influence the child’s development.

The present paper aims to describe the methodology of the Developmental Psychiatry Diagnostic Challenges Study (DePsy), a multi-site, prospective clinical study of six German ECMH clinics (Charité—Universitätsmedizin Berlin, Leipzig University Medical Center, University of Münster, kbo-Kinderzentrum Munich, Vivantes Clinic Friedrichshain, Saarland University Hospital) examining the normal care population of the participating centers. The main focus of the DePsy study is the validation of the DC:0-5. Before outlining the study design and methods, a short review of the history of the DC:0-5 and its predecessors is provided. This study aims to understand the child’s psychiatric disorders in the context of emotionally significant relationships to close caregivers. Thus, a second focus of the study is to examine the child’s relationships with caregivers in relation to parental and environmental risk factors.

### 1.1. DC:0-3 and DC:0-3R

In 1994, a task force of multidisciplinary early childhood clinicians first published a nosology of early childhood disorders for the first four years of life, known as the DC:0-3 [5]. This multiaxial classification system provided infant- and toddler-specific Axis I diagnostic criteria to use as a complement to the International Statistical Classification of Diseases and Related Health Problems (ICD)-10 and Diagnostic and Statistical Manual of Mental Disorders (DSM)-IV. DC:0-3 diagnoses were based on descriptive criteria and on visible and apparent symptoms [8]. Regulation disorders (RD), classified as impaired regulation of neurophysiological, psychomotor, emotional, and behavioral processes, which often are associated with hypersensitivity, impulsivity, irritability, or hyperactivity, sleep and eating problems, were introduced as new diagnoses in early childhood, and became possible precursors to ICD-10 and DSM-IV attention-deficit/hyperactivity disorder (ADHD) and ICD-11 and DSM-5 autism-spectrum disorders (ASD).

The DC:0-3 was the first classification system to present a separate axis for assessing the parent–child relationship (Axis II). In addition, medical and neurological disorders were coded on Axis III according to ICD-10 or DSM-IV, psychosocial stressors were coded on Axis IV, and the child’s emotional developmental status was coded on Axis V. Axis II relationships were assessed using a continuous and a categorical approach: The Parent–Infant Relationship Global Assessment Scale (PIRGAS) was used to quantify the extent of impairment, ranging from 10 (severely impaired) to 90 (well adjusted). Moreover, the quality or type of relationship disturbances were categorized as *overly involved, under-involved, anxious/tense, angry/hostile, mixed, and abusive*. Relationship disorders have been described as both occurring with childhood mental disorders and independently. To date, arguably the greatest merit of the DC:0-3 has been its conceptualization of early childhood psychopathology as a deviant developmental trajectory, and its acknowledgement of the potential distress and functional impairment both within the child and within their environment, and to consider the relational system as a factor that can “*precipitate and perpetuate symptoms, as well as the factors that promote resilience*” [9] (p. 473).

There were also major objections to the DC:0-3, as it was criticized for focusing disproportionately on parental behavior and conceptualizing child behavior as a response to parental behavior. Moreover, the classification of relationships was criticized for being too broad and too imprecise, as a number of criteria were given for each type without specifying how many of them were required for a diagnosis. The PIRGAS had methodological problems as it contained an internal inconsistency in its metric [10]. Despite the great novelty of the introduced relationship assessment, research on PIRGAS and on the quality of relationship disturbances is sparse overall. Few studies have examined the reliability and validity of the PIRGAS as a scale [11,12,13], but there have been almost no attempts to examine the link between relationship impairments and clinical syndromes, or interventions and trajectories.

The revised edition DC:0-3R [6] provided clarifications and specifications of the Axis I criteria to ensure reliability among clinicians and to advance the evidence-based development of the diagnostic system. A key component of the advanced classification was surely the introduction of comorbidity, and the wording of the axes were revised. For Axis II (DC:0-3R), there were minor changes in terms of an expansion of the PIRGAS from a 9-category to a 10-category scale (the new category: documented maltreatment for abuse or neglect), and a checklist for relationship problems. In sum, the revisions of Axis II in the DC:0-3R were considered helpful, but minor, and although some specification of details was provided, the major strengths and weaknesses evident in the DC:0-3 were maintained.

Compared with the research efforts dedicated to validating psychiatric nosology in older children and adults, few studies have addressed the validity of DC:0-3 or DC:0-3R diagnoses, with most of them focusing on Axis I diagnoses and comparing DC:0-3/DC:0-3R and ICD-10 or DSM-IV diagnoses. Existing studies comparing DSM-IV and DC:0-3 diagnoses report concordances between both classification systems, except for RD, which were not included in DSM-IV. Thus, the earliest studies on the DC:0-3 demonstrated a high specificity of symptom patterns for most DSM-IV and DC:0-3 diagnoses apart from regulation disorders [14], but found the DC:0-3 superior in identifying multiple risk factors for problem behavior [15]. Other studies reported a lack of concordance or specificity of RD. Frankel and colleagues [16] compared DSM-IV and DC:0-3 diagnoses in a retrospective study of 177 preschool children. They found groups of disorders with high concordance between the systems as well as those with low concordance, as in the case of disruptive behavior: patients with DSM-IV ADHD diagnoses were most likely assigned to RD in the DC:0-3, with both disorders differing in terms of underlying concepts and treatment. Equit and colleagues’ study [17] showed similar results, comparing ICD-10 and DC:0-3R diagnoses in children in an outpatient early childhood psychiatry clinic. Despite good overall concordances for most diagnoses, a significant part of the sample could only be diagnosed according to ICD-10 (e.g., ADHD or oppositional defiant disorder (ODD)) or to DC:0-3R (e.g., RD), respectively. Skovgaard and colleagues [18] demonstrated that—given a high level of structured information and well-trained raters—both the ICD-10 and the DC:0-3 offered a sufficiently good framework to classify mental health disturbances in the general population of children between 0 and 3 years old. In view of its high reliability in the classification of relationship disturbances, they found the DC:0-3 to be superior to the ICD-10 for this early age group.

### 1.2. DC:0-5

The DC:0-5 [7], i.e., the revision of the DC:0-3R in 2016, became necessary as, after a decade, extensive research had been published on early childhood psychopathology [9,19]. The revision coincided with the publication of the DSM-5 and its attempt to account for developmental differences in symptom expressions in young children. The DC:0-5 was largely aligned with the DSM-5—a fact that brought significant changes to the field. While (unlike the DSM-5) maintaining the axial classification system to emphasize the importance of context for psychopathology in young children, all disorders were re-evaluated for their evidence base and clinical utility. The DC:0-5 provides DSM-5 references for all disorders to create a comprehensive self-standing nosology encompassing all disorders relevant to young children, rather than referring clinicians to other nosologies. Age criteria are used for clinical diagnoses and, where possible, applied to the first two years of life. Specifications are provided for some disorders, and distress and functional impairment criteria have been introduced for each disorder to distinguish between mental disorders and transient phenomena. However, the major changes are certainly the expansion of the age range from 3 to 5 years, as indicated in the title, as well as the introduction of newly defined disorders, e.g., neurodevelopmental disorders (e.g., Infantile Hyperactivity Disorder, Early Childhood Atypical Autism Spectrum Disorder), the Early Childhood Relationship Disorder, and the inclusion of disorders defined in the DSM-5 but not in the DC:0-3R, e.g., ADHD, disorder of dysregulated anger and affect (DDAA), and of disorders defined differently from the DC:0-3R (e.g., eating disorders). The clinically highly differentiating RDs and feeding disorders were replaced by Sensory Overstimulating and Understimulating Responsivity Disorder, and Overeating and Undereating Disorder, respectively.

Because the relationship between the primary caregiver and the young child is often the focus of clinical assessment and intervention, and because, as recent research has shown, the extended environment of family relationships can also have an impact on child development (e.g., co-parenting [20]), the relationship axis was significantly modified. For Axis II (DC:0-5), a simplification of the relationship assessment was implemented so that both the overall adaptation of the infant/young child’s primary caregiving relationships (Part A of Axis II) and the infant/young child’s caregiving environment (Part B of Axis II) can be assessed at four levels: Level 1—*Well adapted to Good Enough Relationships* (relationships that are not of clinical concern), Level 2—*Strained to Concerning Relationships* (relationships where monitoring is indicated and intervention may be required), Level 3—*Compromised to Disturbed Relationships* (the relationship disturbance is within the clinical range, and intervention is indicated), and Level 4—*Disordered to Dangerous Relationships* (intervention is not only required but urgent due to the severity of the relationship impairment). Both axes feed into the clinical formulation and determine the type and intensity of intervention needed.

In providing a framework for the standardized psychiatric assessment of psychopathology in infants, toddlers, and preschool children [4], the DC:0-5 is currently widely used by clinicians working in the field of infant and early childhood mental health [21]. In contrast to its clinical utility, studies empirically corroborating the DC:0-5 are scarce. To the best of our knowledge, Hussong and colleagues’ [8] is the only existing study on the validity of the DC:0-5. The authors compared DC:0-5 and ICD-10 diagnoses in a group of consecutively presenting preschool children (*n* = 176) from a German psychiatric outpatient clinic. In the study, most of the children included received DC:0-5 or ICD-10 diagnoses, with the DC:0-5 more comprehensively representing behavioral difficulties in infancy.

### 1.3. Emotional Availability, Parental, and Environmental Risk Factors

By classifying the quality of primary caregiver relationships, the DC:0-5 acknowledges the relevance of close relationships in the emotional development of early childhood. Attachment research, which provides the most comprehensive evidence on the role and specificity of close relationships in childhood, has inspired approaches to describing and assessing the characteristics of dyadic relationships and their quality, a prominent one being the concept of emotional availability (EA) [22]. EA can be defined as “the capacity of a dyad to share an emotional connection and to enjoy a mutually fulfilling and healthy relationship” [23] (p. 1).

EA represents a multidimensional approach to characterize the interactions in dyadic relationships, and it has inspired a coding system to assess the overall affective quality of the caregiver–child relationship: the Emotional Ability Scales (EA Scales). Evaluating the EA between a caregiver and child on six dimensions, the EA Scales can provide a means to validate Axis II of the DC:0-5. Furthermore, as EA has been shown to play a significant role in the child’s psycho-emotional development (for an overview of findings see, e.g., [24]), it is likely that psychiatric disorders in early childhood are associated with impaired EA in the relationship between the child and its primary caregiver(s). But as the EA approach has hardly been incorporated in research in early childhood psychiatry (fan exception: [25]), little is known about the associations between EA and specific disorders in early childhood. DePsy aims to contribute to integrating EA research into early childhood psychiatry.

Finally, EA in the relationship between child and caregiver represents a possible pathway through which parental and environmental risk factors could affect the child. Two risk factors are examined in more detail in the DePsy study: adverse childhood experiences (ACE) and media use.

#### 1.3.1. Adverse Childhood Experiences (ACE)

An adverse impact of trauma, family conflict, loss of a caregiver, or abusive experiences on emotional development in early childhood, has been postulated in several large studies [26]. Overall, a considerable number of studies have pointed out the negative impact of childhood adversities (CA), such as physical and sexual abuse, neglect, parental loss or family conflict, on the development of psychopathology in adulthood, emphasizing the need to consider ACEs as an influential factor in models examining parents and children. Two-generational studies have revealed that a significant proportion of abused parents transmit this history of abuse to their offspring [27]. The experience of trauma, especially in the form of sexual abuse and physical abuse, but also emotional neglect in childhood or adolescence, has been postulated to play a major role in parenting [28]. In parents, not only psychopathology, but also neurobiology [29], as well as psychophysiology and attachment, have been described as altered after a history of early life stress, such as childhood abuse [30]. Thus, intergenerational transmission has been postulated to occur via several pathways [31]. Among other factors, it has been assumed that a longer-term experience of stress or abuse during the unfinished development of regulatory and coping mechanisms might lead to impairment of behavioral control, leading among other harmful effects, and altered parenting behavior. Endeavors of prevention have focused on the transgenerational ‘cycle of abuse’, i.e., the possibility that experiences of childhood abuse could interfere with later parenting behavior, the perception of one’s own parenting abilities as well as their perception of and interaction with the child. Therefore, it is essential to analyze adverse childhood experiences (ACE) in parents of infants, toddlers, and preschoolers presenting at early childhood mental health clinics.

#### 1.3.2. Early Media Use and Psychopathology in Children Aged 0 to 5 Years

Research on media use in young children shows the wide availability of media devices and extensive usage times. Young children have not yet fully developed their socio-emotional abilities, while they exhibit a particularly high neuronal plasticity. This mismatch represents the particular risk of media use for further child development [32]. Previous studies have shown a range of negative effects due to excessive early media use, especially on children’s socio-emotional development [33,34,35]. Excessive media exposure was shown to be associated with emotional dysregulation [32,36], emotional and conduct problems, hyperactivity, and inattention [37]. In infants and preschoolers, several other studies found evidence of the harmful effects of television exposure with respect to emotional problems [38], externalizing behavior [39], hyperactivity, and inattention [33,40], as well as developmental problems and oppositional behavior [41]. Additionally, children with mental disorders, such as ASD, are exposed to higher levels of media use [42,43,44].

Within the interaction theory of problematic media use in childhood [45], Trumello and colleagues [46] found that maternal, but not paternal, EA predicted adolescent internet addiction: lower levels of emotional quality in the maternal relationship were associated with higher levels of internet addiction. The same was true for the quality of parent–child attachments and relationships. A review [47], as well as two longitudinal studies, reported negative associations between the quality of parent–child relationships [48] or family atmosphere [49] and problematic gaming. The use of smartphones in the family context, especially in the care situations of young children, has been shown to reduce parental sensitivity, communication, and affect exchange [50,51,52,53,54,55,56].

For elementary school children, teenagers, and young adults, many studies describe an association between mental disorders (e.g., depression, anxiety, ADHD, ASD) and gaming disorder, internet addiction, problematic internet use, or other dysfunctional uses of digital media [57,58,59,60,61,62,63,64,65]. A literature search showed almost no comparable results with dimensional approaches or categorical ICD-10 diagnoses for the age group of 0–5 years, nor with the classification systems of the DC:0-3, DC:0-3R, and DC:0-5, as media-associated disorders are not yet included in them. Given the wide availability of media devices and extensive usage times among young children, a consideration and implementation of the “Dysfunctional Media Use in Preschool Age” disorder will need to be researched and verified for a future version of the DC:0-5.

## 2. Study Aims and Hypotheses

The **main aim** of this study is to contribute to the empirical validation of the DC:0-5 classification, focusing on its use in routine clinical practice.

First, the validity of DC:0-5 Axis I diagnoses will be tested by comparing them with diagnoses according to the ICD-10, which is commonly used in Germany, as well as with clinical questionnaires. Since the DC:0-5 provides a comprehensive psychiatric classification system for early childhood and preschool, we expect that the proportion of children whose symptomatology cannot be assigned to a specific psychiatric diagnosis to be higher for the ICD-10 than for the DC:0-5. We further expect a missing or non-specific ICD-10 diagnostic assignment (classification as “other” or “unspecified”) especially for children whose symptomatology is coded as a Sensory Processing Disorder, Overactivity Disorder of Toddlerhood, Inhibition to Novelty Disorder, Disorder of Dysregulated Anger and Aggression of Early Childhood, Excessive Crying Disorder, or Relationship Specific Disorder of Infancy/Early Childhood in the DC:0-5. In clinical questionnaires, children with specific disorders in the DC:0-5 are expected to score in the clinical range on the corresponding scales of the Child Behavior Checklist (CBCL)1½-5 [66] (ADHD: CBCL-Scale *Attention Problems*; Anxiety and Mood Disorders: CBCL Scale *Anxious/Depressed*; Disorder of Dysregulated Anger and Aggression: CBCL-scale *Aggressive Behavior*; Sleep Disorders: CBCL Scale *Sleeping Problems*).

Second, new Axis I disorders introduced in the DC:0-5 (e.g.,: Relationship Specific Disorder of Infancy/Early Childhood; Disorder of Dysregulated Anger; and Aggression of Early Childhood) will be described in terms of symptoms, comorbidities, and course.

Third, Axis II—*caregiving dimension*—diagnoses will be validated by the EA Scales and by self-ratings of parental stress. Axis II diagnoses—the *caregiving environment dimension*—are validated by clinical ratings of family adversity. We expect a compromised EA and elevated parental stress for caregivers of patients with assignments of Level 2 or higher on the *caregiving dimension* of Axis II of the DC:0-5. An elevated family adversity index is expected for patients with assignments of Level 2 or higher on the *caregiving environment dimension* of Axis II of the DC:0-5.

The **second aim** of the study is to describe cross-sectionally a typical care population of six ECMH clinics in Germany. Presenting symptoms, diagnoses, demographic characteristics, and the course of symptoms, as well as clinical outcomes after treatment, will be examined. This aim also includes an exploratory longitudinal evaluation of a sub-sample of the clinical population enrolled in the study. Over a period of at least six months, we will examine the stability of the diagnoses assigned at the first visit, the course of the children’s symptoms, implementation of the therapeutic measures recommended, and treatment satisfaction of the families enrolled in DePsy.

The **third aim** of the study is the exploratory investigation of how parenting stress and emotional availability are related to the assigned Axis I diagnoses of the DC:0-5.

A **fourth aim** is the examination of the impact of parental ACEs on the child’s psychopathology and on the relationship between the child and primary caregiver. One hypothesis of this study is that parental ACE scores are associated with infant psychopathology (elevated scores on the CBCL scales for *total problems*, *internalizing*, and *externalizing*). Furthermore, we expect a negative association between parental ACE scores and the parental scale scores on the EA Scales.

A **fifth aim** of the study is to explore the relationship between mental health disorders (e.g., externalizing and internalizing behavioral problems, ADHD, ODD, ASD) in children aged 0 to 5 years old and early media use. We expect an influence of child mental health problems (categorically and dimensionally considered) and parental media use (media use times and problematic internet use) on child media use (disorder criteria and use times).

A **sixth aim** of the study is to investigate the parent–child interaction in relation to parental and children’s media use. Increased media use time or problematic internet use by parents is expected to have a negative impact on parent–child interactions.

## 3. Methods

### 3.1. Study Setting

The Developmental Psychiatry Diagnostic Challenges Study (DePsy) is a multi-center study across six university hospitals in Germany.

#### 3.1.1. Charité—Universitätsmedizin Berlin

The ECMH clinic at the Charité—Universitätsmedizin in Berlin is part of the outpatient Center of the Department of Child and Adolescent Psychiatry at Charité Universitätsmedizin Berlin (Berlin, Germany). The ECMH clinic offers high-frequency outpatient treatment for infants, toddlers, and preschool children, with around 300 cases per year. The clinic is located in a large metropolitan region with a high proportion of families with low socioeconomic status and high rates of immigrants.

#### 3.1.2. kbo-Kinderzentrum (“Children’s Center”) Munich

The kbo-Kinderzentrum has both an inpatient as well as an outpatient clinic. For the present study, only patients of the inpatient clinic will be recruited. The Clinic of Social Pediatrics at the kbo-Kinderzentrum Munich is the largest social pediatric clinic in Germany. Yearly, 800 children and their parents are treated in the inpatient clinic. Patients are children from 0–18 years who stay at the inpatient unit with their parents for at about 3–8 weeks. Besides a large group of children with developmental disorders, one main focus is the treatment of infants/toddlers with Excessive Crying, Sleep, and/or Eating Disorders, as well as Relationship Disorders.

#### 3.1.3. Leipzig University Medical Center

The specialized parent–child service at the Leipzig University Medical Center, Department of Child and Adolescent Psychiatry, Psychotherapy and Psychosomatics focuses on infants, toddlers, and preschool children with mental disorders. The main focus of the in- and outpatient clinic is the treatment of early onset regulatory and interaction disorders. Yearly, 60 infants, children, and parents are treated in the inpatient and 800 in the outpatient setting of the parent–child service.

#### 3.1.4. Münster University Medical Center

The specialized outpatient clinic for young children and preschoolers and their parents, as well as the family day clinic for preschool children, are part of the Clinic for Child and Adolescent Psychiatry, Psychosomatics, and Psychotherapy at the University Hospital Münster. In these clinics, 210 outpatients are seen annually, and inpatient treatment is provided for children with mental or psychosomatic illnesses, with the close therapeutic involvement of their parents. About 120 inpatients are seen annually. In collaboration with the Clinic for Mental Health, an interdisciplinary outpatient clinic for Mental Health for parents and children during pregnancy, childbirth, and infancy is also offered. Additionally, as a joint offering of both clinics, the Parents and Baby Day Clinic is expected to open at the end of 2023. This clinic will provide parallel treatment for mentally ill parents and their infants who are also suffering from mental or psychosomatic illnesses.

#### 3.1.5. Saarland University Hospital

The special outpatient clinic for infants, small children, and preschool children with mental disorders, located at Homburg, is part of the Department of Child and Adolescent Psychiatry, Psychosomatics, and Psychotherapy at Saarland University Hospital. It has existed for 20 years and is closely linked to an associated child psychiatry parent–child ward. Each year, around 600 families are treated.

#### 3.1.6. Vivantes Klinikum Berlin

The Vivantes Clinics for Child and Adolescent Psychiatry offer 15 day care treatment places for small and preschool children with psychiatric disorders at the Berlin Neukölln site. Individual places are available for therapeutic parent–child treatment. After an acclimatization phase, the children aged one year to school entry spend the day in the KiTa-like children’s group. Psychotherapeutic work takes place several times a week in the parent–child setting. Furthermore, the children are cared for by curative educators and various specialized therapists in individual and group appointments. The outpatient clinic for toddlers and preschoolers is open to children from birth until they start school. Currently, approximately 480 patients are seen per year, with a frequency ranging from one to 12 appointments per quarter. In addition to case management by a child and adolescent psychotherapist, medical and specialist therapeutic diagnostics take place as needed.

### 3.2. Study Design, Eligibility Criteria, and Recruitment

#### 3.2.1. Study Design

DePsy is an observational study of children undergoing outpatient or inpatient treatment at the ECMH clinics of the six above-mentioned DePsy sites. Table 1 provides an overview of the complete study design, including methods and instruments. As the study is closely based on usual clinical care, it will analyze the data collected during routine clinical practice, supplemented by a small number of questionnaires tailored to DePsy. DePsy employs both a cross-sectional as well as a smaller longitudinal design including study entry (T0) and one follow-up time point (T1). The T1 follow-up is not mandatory for all subjects enrolled, but will only take place if the children present again for examination in the clinic within a period of 9–15 months since study entry.

#### 3.2.2. Eligibility Criteria

Children from the six participating ECMH clinics, aged 0–5.9 years, and entering the ECMH services are eligible for inclusion in the DePsy study. The child as well as one adult caregiver are enrolled in the study. The participating caregiver is a parent in most cases; for children living in residential youth care facilities or in foster families, caregivers could be an educator or a foster parent. Participation further requires the informed consent of the legal guardian(s) of the children involved and, if different, also of the participating caregiver(s). A further requirement for participation is sufficient competence of the German language on the part of the caretaker in order to complete the assessments.

#### 3.2.3. Recruitment

Participants are enrolled by primary clinicians during the clinical consultation. All families who meet the eligibility criteria are approached by the primary clinician at each of the corresponding ECMH clinics and invited to participate.

### 3.3. Data Collection

#### 3.3.1. Baseline Clinical Assessment: T0

Outpatients who are presented by their caretakers for treatment at the clinic for the first time will be sent an initial intake form in advance, in which socio-demographic and anamnestic information about the child’s development is requested. This form will be completed by the caretaker.

A comprehensive assessment of the child’s psychiatric status as well a review of the child’s presenting symptoms and behavioral problems are conducted during an initial clinical interview, which lasts between 60 and 90 min and takes place with the caregiver and child. In addition to the initial clinical interview, a second appointment is usually held to complete the family and developmental history, which only takes place with the caregiver to allow for open conversation. The initial clinical interview will be conducted by the responsible primary clinician, either a clinical psychologist or a medical doctor, both with profound experiences in infant mental health. Furthermore, interviews with their preschool teachers will be conducted in order to obtain information on the child’s behavior in preschool. In addition to the initial clinical interview, the caregiver completes questionnaires on the child’s psychopathology (CBCL 1½-5 [66]/Questionnaire for Crying, Feeding, Sleeping (QCFS) [67]), parental stress (German version of the Parental Stress Index “Eltern-Belastungs-Inventar” EBI [68]), media use (Menu-Juki 0-5 [69]), and ACE [26]. Parent questionnaires on child emotional or behavioral problems may be subject to response bias [66], and studies have to find ways to mitigate this issue. Before caretakers complete the questionnaires, the responsible clinician will discuss possible questions, explain confidentiality, and point out to the caretakers that the main purpose of these instruments is to gain a better insight into the child’s problems and not to evaluate and judge parenting. Based on the information from the initial clinical interview, further appointments for follow-up assessments will be arranged based on clinical indication. A physical examination of the child will be performed as standard procedure, except in cases where a pediatric screening has taken place shortly beforehand. If the developmental status is part of the question leading to the child’s presentation or if there are indications of a developmental delay, developmental testing will be initiated. In the case of children under 36 months, the Bayley scales of the Infant and Toddler Development Screening Test-Third Edition (BSID-III) [70] will be administered. For children over 36 months, an intelligence test—the Snijders Ooomen Nonverbal Intelligence Test (SON-R 2-8 [71]) or the Wechsler Preschool of Primary Scale of Intelligence, Fourth Edition (WPPSI-IV [72])—will be conducted. If there are signs of delays in language or motor development, the child will be presented to an occupational or speech therapist for an assessment of language and/or motor development.

For all children younger than 36 months, and for patients exposed to a troubled caregiver–child relationship, a standardized video-based assessment of the caregiver–child interaction will be initiated. The caregiver–child interaction is observed in a video-taped session lasting 10 min. The caregiver and child will be placed in a laboratory playroom and instructed to play as they normally would in a free play situation. The quality of the interaction will be analyzed using the EA Scales [73].

ICD-10 and DC:0-5 psychiatric diagnoses are assigned based on the information gathered during the clinical assessment process. Diagnoses are decided on by mutual consensus at regularly occurring diagnostic case conferences at each of the study sites. The diagnostic case conferences are attended by the primary clinician and a multidisciplinary team, consisting of clinical psychologists, child psychiatrists, as well as by the senior physician in charge. The members of the clinical team are involved in the study in different ways, all part of the team, but not all team members will be blinded to the study objectives. Inter-rater reliability testing of psychiatric diagnoses will be conducted in 15% of the cases by reviewing assessment results and medical records. The reliability check will be carried out by a clinical psychologist or child psychiatrist at each site who has not been involved in previous assessments and who is therefore blind to the diagnoses. To ensure the reliability of the diagnoses across sites, regular multisite diagnostic case conferences will be held involving case managers from all the participating clinics. Cases of patients where the diagnostic process has not led to an unequivocal diagnosis at a study site will be discussed at this multisite diagnostic case conference.

Patients who have previously received a psychiatric evaluation at the outpatient clinic and who are still in treatment will undergo a shorter diagnostic process. In these cases, acute emotional and behavioral problems and a current psychiatric status will be assessed in a clinical interview conducted by the primary clinician. The primary clinician will be responsible for updating and completing the socio-demographic information as required by the study protocol. The caretaker will be asked to complete the study questionnaires. On re-presentation, DC:0-5 and ICD-10-diagnoses will be assigned according to the procedures described above. Further assessments of the child’s developmental status or video-based interaction assessments will be initiated if clinically indicated.

Patients treated as full-time or part-time inpatients at study sites with inpatient services have previously had one or more outpatient appointments where the indication for inpatient or day care treatment was made. The child’s day clinic treatment takes place two or three days a week over a period of an average of 12 weeks, during which a caregiver is always present. Some patients receive a second, on average 4-week, day care treatment interval (booster therapy). Full inpatient treatment lasts about 3–6 weeks; one caregiver is admitted as well. The participants entering the study as full or day hospital patients will fill out the study questionnaires as part of the treatment. Assessments of physical and developmental status, video-based interaction assessments, and the assignment of psychiatric diagnoses are similar to the procedures described for outpatients.

#### 3.3.2. Follow-Up Clinical Assessment: T1

Patients who are re-presented at the outpatient clinic within 9 to 15 months from the baseline clinical assessment T0 are eligible for a clinical follow-up assessment (T1). The follow-up assessment includes a psychiatric assessment with the assignment of ICD-10 and DC:0-5 diagnoses following the steps outlined above (see Section 3.3.1). Further assessments (developmental/language/motor tests; physical examinations; video-based interaction assessment) will be initiated if clinically indicated for further psychiatric assessment. Furthermore, any interventions initiated since T0 are collected, and the caretaker will complete questionnaires on child psychopathology (CBCL 1½-5; QCFS) and on treatment history (FraBeHa [74]).

### 3.4. Measurements

#### 3.4.1. Sociodemographic Characteristics

Sociodemographic variables referring to the family and to the child’s development as well as information on medical history and on previous or ongoing youth welfare interventions will be obtained from the patient’s medical record. Information on the child’s physical development (height, weight) will be collected as part of the physical examination. The psychiatric ICD-10 and DC:0-5 diagnoses will be obtained from the patient’s record and from the medical report.

#### 3.4.2. Cognitive Measurements

For patients younger than 36 months, the cognitive scale from the Bayley scales of Development Third Edition (Bayley-III) will be utilized as an assessment of the child’s cognitive status [70]. For the German version of the cognitive scale of Bayley-III, good reliability (mean reliability: 0.82) was reported. A study on convergent validity for the original U.S. version of the instrument found high correlations between the cognitive scale of the Bayley-III and the full-scale IQ of the WPPSI-III (0.79) [70]. For patients 36 months or older, intelligence will be assessed using an age-appropriate standardized intelligence test, either the SON-R 2-8 [71] or the Wechsler Preschool and Primary Scale of Intelligence (WPPSI), Fourth Edition [72]. For both measurements, the total IQ will be utilized for the study. Both measurements are well established and broadly validated intelligence tests with excellent psychometric properties (SON-R 2-8—reliability 0.89–0.93; construct validity: the correlation between SON-R 2-8 and Wechsler Nonverbal Scale of Ability (WNV) is 0.74 [71]. WPPSI-IV—reliability 0.93–0.95; construct validity: the correlation between WPPSI-IV and Wechsler Intelligence Scale for Children (WISC)-V is 0.69 [72]).

#### 3.4.3. Emotional Availability

The Emotional Availability Scales, 4th edition [73] will be used to analyze the videotaped caretaker–child interaction. The EA Scales are a well-established assessment system of the dyadic interaction between a caregiver and a child, focusing on the emotional quality of the relationship. The EA Scales contain six dimensions, four of which—(1) sensitivity, (2) structuring, (3) non-intrusiveness, and (4) non-hostility—are assigned to the caretaker and two—(5) responsiveness and (6) involvement—to the child. All dimensions are coded on a Likert-type ordinal scale ranging from 1 (lowest) to 7 (highest). Previous studies have reported good psychometric properties for EA Scales. In a review on EA Scales, Biringen and colleagues [23] reported reliability scores ranging between 0.76 and 0.96 for the EA subscales. Validity studies on EA Scales mostly examined associations between the EA Scales and indicators of attachment, such as the Attachment Q-Sort. Studies found convergent validity between attachment measures and the EA Scales, especially for the scales of caretaker sensitivity and child responsiveness and involvement [22,23]. EA scales will be coded by clinical psychologists who have been trained in the EA Scales and approved as reliable by the author of the EA Scales.

#### 3.4.4. Child Psychopathology

The Child Behavior Check List (CBCL) 1½-5 will be administered for the assessment of psychopathology in children 18 months of age or older. Psychopathology in children younger than 18 months will be measured with the Questionnaire for Crying, Feeding, and Sleeping (QCFS). The CBCL 1½-5 is a standardized and widely used parent questionnaire assessing behavioral and emotional problems in children between 1.5 and 5 years. The German version of the CBCL 1½-5 [66] includes 100 items that are rated on a three-point Likert-scale (0 ‘not true’, 1 ‘somewhat or sometimes true’, 2 ‘very often true or often true’). The CBCL 1½-5 contains a total problem score as well as seven subscales of which four are grouped under the internalizing scale (Emotionally Reactive, Anxious/Depressed, Somatic Complaints, Withdrawn), two are grouped under the externalizing scale (Attention Problems, Aggressive Behavior), and one scale covering disturbances to sleep (Sleep Problems). The CBCL 1½-5 has excellent proven reliability and acceptable validity in research and clinical care [66]. The caretaker questionnaire QCFS [67] dimensionally assesses regulatory problems in infancy. This 49-item questionnaire includes three scales: (1) crying, fussing, and sleeping, (2) feeding, (3) dysfunctional parental co-regulation, and a total score. Satisfactory reliability has been reported in previous studies [67,75].

#### 3.4.5. Parenting Stress

The German version of the Parenting Stress index (EBI) [68] will be used in order to assess the subjective burden of the caretaker in parenting. The Parental Stress Index is a well-established 48 item self-report questionnaire for caretakers that focuses on parental stress and can be utilized as a screening tool for dysfunctional parent–child interactions. The instrument comprises a total score and two domain subscales. The parent domain covers deficits in parental functioning including upbringing and care, and the child domain assesses demands resulting from characteristics of the child’s behavior. Good psychometric properties have been reported for the EBI with reliability scores ranging from α = 0.95 for the total score and α = 0.93/0.91 for the domain scores [68]. The validity of the instrument has been examined in several studies that have reported substantial associations between EBI scores and various indicators of parental stress.

#### 3.4.6. Media Use: Menu-Juki 0-5

The Menu-Juki 0-5 parent questionnaire on media use in young children aged 0–5 years is a parent questionnaire assessing the availability, contexts, and average per day duration of electronic media use (e.g., television, computer/laptop, smartphone, smartwatch, tablet, game console, and television) in children aged 0–5 years as well as the family contexts of use [69]. The questionnaire contains five items used to record the parent’s problematic internet use, as well as further questions to record their media consumption (time, occasions, reasons for use). In addition, it is assessed whether the parent or guardian has one or more social media accounts (e.g., Facebook, Instagram, Snapchat, TikTok).

#### 3.4.7. Treatment History

The FraBeHa questionnaire on treatment history is a questionnaire to assess intervention programs, for families with children aged 0–5 years, in which the families have participated or are currently participating [74]. The questionnaire asks about a broad spectrum of possible interventions in which the young child (11 items), but also the legal guardians (9 items), may have participated (yes/no answers) as well as the reasons for treatment and the start and end points. In addition, satisfaction with the effectiveness of the treatment is recorded for each intervention on a six-point scale. The questionnaire includes 9 items for the legal guardians (“Help for the family or treatment of the parents”).

#### 3.4.8. Adverse Childhood Experiences

The Adverse Childhood Experience (ACE) questionnaire is a short screening tool for adults adapted from the work of Kaiser Permanente and the Centers for Disease Control and Prevention [26]. The instrument has been compiled by the Office of the California Surgeon General and Department of Health Care Services in consultation with the California Surgeon General’s Clinical Advisory Subcommittee. DePsy uses ACE-D, the authorized German translation. The questionnaire comprises 10 items on one scale, and it takes about 5 min to complete. Psychometric characterization of the German version has been provided [76].

### 3.5. Ethical Approval

Ethical approval was obtained by the ethics committees of the corresponding university medical centers: Charité Berlin (protocol code EA2/005/21; date 29 January 2021), University of Saarland (Ärztekammer des Saarlandes Bu 15/22; date 31 January 2022), University of Leipzig (131/22-lk; 16 May 2022), University of Münster (2021-526-b-S; 21 September 2021), and Munich (669/21 S-KK; date 24 November 2021). Patients will receive verbal and written information about the content and aims of the study and consent forms from the primary clinicians. Participation in the study is voluntary and requires written consent from the legal guardian(s). Consent can be withdrawn at any time without consequences for the patients and their families. The study procedures follow the Declaration of Helsinki.

### 3.6. Data Analytic Procedures and Sample Size

Data on the presenting population with regard to age, sex, symptomatology, referring institutions, and diagnoses will be analyzed with descriptive statistics. Descriptive statistics and phi-correlations will be used to compare the ICD-10 and DC:0-5 diagnostic systems with regard to the diagnostic groups assigned. Chi-square tests will be performed in order to test the hypothesis that a psychiatric assessment according to ICD-10 leads to more non-specific diagnoses in infants or preschool children compared to a diagnostic assessment with the DC:0-5.

For the validation of Axis I DC:0-5 diagnoses with clinical questionnaires, descriptive statistics will be used to determine the proportion of children in different diagnostic groups who fall within the clinical range of the corresponding subscales of the CBCL 1½-5 and QCFS clinical questionnaires. In addition, we will compare the proportion of children with DC:0-5 diagnoses who are in the clinical range of the corresponding subscales of CBCL 1½-5 and CFS with the proportion of children in an age-matched clinical control group via Chi-square tests.

To validate the *caregiving dimension* of Axis II of the DC:0-5, we will compare parental stress and emotional availability of caregivers with scores ≥ 2 on the *caregiving dimension* of Axis II of the DC:0-5 to caregivers with scores < 2. For the validation of the *caregiving environment dimension* of Axis II of the DC:0-5, we will compare the family adversity index between both groups. Intergroup differences will be analyzed with *t*-tests and nonparametric tests, such as the Mann–Whitney U-test.

To exploratorily investigate how parenting stress and emotional availability predict the likelihood for the child’s being in each Axis I diagnosis category or more general categories, such as internalizing and externalizing disorders, we will use logistic regression analysis. To exploratorily determine if there are differences in parental stress and emotional availability across Axis I diagnoses of the DC:0-5, we will use a multivariate analysis of variance (MANOVA).

To address the question of the impact of the parental ACE scores on infant behavioral development and emotional availability, we will test whether the parental ACE score and CBCL Total Problem Score (as an indicator for infant behavioral development) on the one hand, and EA Scales scores and ACE on the other hand, are related using Pearson’s r. If the requirements for Pearson’s r are not met, Spearman rank correlations will be calculated.

For the expected influence of child mental health and parental media use on child media use, multiple regression analysis will be performed to examine whether child age, child sex, child mental health disorder (using the CBCL Total Problem Score and the dimension of the externalizing versus internalizing CBCL Score), as well as the level of problematic internet use (symptom criteria of the primary caregiver) and media use time of the primary caregiver, influence child media use time and mental health disorder criteria. For the expected impact of the amount of parental media use time or problematic internet use on parent–child interactions, we will capture the quality of parent–child interactions using six EA Scales scores (sensitivity, non-intrusiveness, structuring, non-hostility, responsiveness, involvement). We will test, via multiple regression, how each of these EA Scales scores can be predicted according to child age, child sex, child mental health disorder (using the CBCL Total Problem Score and the dimension of the externalizing versus internalizing CBCL Score), as well as the level of problematic internet use (symptom criteria of the primary caregiver) and media use time of the primary caregiver. Spearman rank correlations will be used to determine whether there is a correlation between the six EA Scales scores and the child’s media usage time on the one hand, and the child’s degree of problematic internet use on the other.

To determine the required sample size, we focused on the main aim of the study: the empirical validation of the DC:0-5. Sample size calculation for the empirical validation of the DC:0-5 posed challenges for us. Regarding the conflicting methodological approaches to validating diagnostic systems, the criteria used for sample calculation for such studies are controversial [77]. Validation studies for the DC:0-5 on which sample size calculation could be based are still lacking. There are empirical validation studies for the DC:0-3 or DC:0-3R with similar aims and designs to our study, which compared the DC:0-3 and ICD-10/DSM-IV in clinical samples [16,17,78]. However, regarding the considerable differences between the DC:0-3 and DC:0-5 in structure and scope, we decided not to use the indices reported there for the sample calculation of our study, but rather to determine the sample size a priori. In order to detect a small-to-medium association (ω = 0.2) in Chi-square contingency table tests with a type I error level of α = 0.05 and sufficient power (≥80) (as proposed in our second research aim), a sample size of 200 is required (calculated using G*Power^®^). A sample size of 200 will also have a sufficient power of >0.80 to detect a small-to-medium effect size (d = 0.4) in a two-sided test comparing different means with α = 0.05.

### 3.7. Dissemination

Study results will be published in international and national scientific journals and will be presented at scientific conferences. Reporting of the results will follow the STROBE guidelines (https://www.strobe-statement.org/) (accessed on 21 October 2023). To avoid publication bias, we will follow a transparent and comprehensive publication strategy, including the reporting of negative results. Furthermore, we will use meetings and information channels of relevant national and international scientific societies (e.g., Deutsche Gesellschaft für Kinder- und Jugendpsychiatrie, Psychosomatik und Psychotherapie (DGKJP), World Association for Infant Mental Health (WAIMH)) to disseminate the results of our study.

## 4. Discussion

Our protocol addresses several topics pertinent to improving mental health care in infancy and early childhood. Due to its multi-site design, the study will provide comprehensive information on the clinical population of ECMH clinics in Germany, including data on presenting symptoms, referring institutions, the distribution of psychiatric disorders, and the involvement of the patients in different care systems. The collection of a sufficiently large sample of children with mental health issues from different ECMH clinics across Germany, facilitated by the multi-site design, is an initial strength of the study. The study can provide an overview of the present status of psychiatric care in infancy and early childhood, thereby highlighting possible gaps in care.

To the best of our knowledge, DePsy is the first study to focus on an extensive validation of the DC:0-5 in a clinical sample. The DePsy study takes a comprehensive approach to validating the DC:0-5, including both Axis I and Axis II, and it encompasses multiple methods, which is a second strength of the study. In addition to a comparison between DC:0-5 and ICD-10 psychiatric diagnoses, which follows the approach of previous studies [8], questionnaires, observational procedures, and sociodemographic data will be used to test the validity of Axis I and Axis II diagnoses.

Despite the relevance of media in shaping children’s environments, research on the role of media use in early childhood and its interaction with socio-emotional development is scarce, especially in clinical populations. Research on intergenerational transmission has revealed the effects of parental early life stress on the second generation, so that parental childhood adversities should be taken into account in clinical care in ECMH clinics. That both contextual aspects are considered in DePsy can be seen as a third strength of the study.

Based on the experience of a large number of practitioners, and providing guidelines for the clinical assessment in early childhood, the DC:0-5 is rooted in clinical practice. In line with this clinical orientation, maximum proximity to clinical care is a leading principle of the DePsy study. The majority of the information used in DePsy comes from routine clinical care, and the selection of the assessments is based on clinical indication.

Accordingly, although the study has strengths, it also has notable limitations, most of which arise from its nature as an observational clinical study. The first set of limitations is related to the sample. Because the recruitment of the DePsy study is limited to patients from ECMH clinics, it does not provide a representative sample of preschool children, so that its results do not allow conclusions to be drawn about the prevalence or course of specific disorders in the general population. This limitation also pertains to the part of the study devoted to media use. As the results will be obtained from a clinical sample, they cannot be extrapolated to the general population. Furthermore, as participating clinics will differ in their respective patient populations due to specialization and setting, samples might be heterogeneous with respect to socio-economic background or age, which could be considered a further limitation of the study. On the other hand, such heterogeneity may enhance the external validity and generalizability of the findings.

A second set of study limitations arises from the use of clinical data. The study uses clinical diagnoses, which may affect reliability. A higher degree of standardization, and therefore potentially higher reliability, could have been achieved by using structured interviews to assign psychiatric diagnoses. Furthermore, as the study is the result of a joint effort of clinicians and is very close to clinical practice, it is not possible to blind all members of the clinical team to the study objectives and hypotheses. This is a further limitation of the study. However, DePsy follows the procedures in the ECMH clinics as closely as possible and aims at optimizing the assignment of clinical diagnoses. ICD-10 and DC:0-5 diagnoses will be obtained through the consensus of clinical psychologists or medical doctors with many years of clinical experience in the field. Regular inter-site clinical case conferences will help to ensure the reliability of psychiatric diagnoses across sites.

A final set of limitations relates to the design of the study. Some of the research hypotheses include associations between variables that cannot be interpreted as causal relationships due to the cross-sectional design of significant portions of the study. In addition, the longitudinal part of the study lacks a control group, which also hampers causal inferences about the potential effectiveness of therapeutic interventions. Due to the observational nature of the study and its dependence on the clinical indication of visits and assessments, the longitudinal follow-up will be conducted only in a subsample of patients, which can lead to a selection bias and a significant reduction of the follow-up sample. In addition, the follow-up period is limited to 9–15 months. Therefore, the results of the study will not provide information about the medium- or long-term course of the disorders studied, which would need to be accomplished in future studies. Nevertheless, this study can provide helpful information that can be used to design more definitive studies that can test longer duration outcomes and causal inferences that are informed by our cross-sectional results and medium-term longitudinal findings which are lacking a control group.

## 5. Conclusions

Infant and toddler psychiatry is a comparatively young specialty, both in terms of clinical services and care, and in terms of research. Given the relatively high prevalence of mental disorders in this age group, much remains to be done to address the unique manifestations of psychopathology in young children in the midst of their development and relationships, as well as the special needs of this particularly vulnerable patient population.

The present overview of clinical care across the six participating ECMH clinics can help standardize and optimize mental health care with respect to diagnostic assessment, clinical formulation, and therapeutic interventions for infants, toddlers, preschool-aged children, and their families. A high level of expertise is required for a developmentally sensitive assessment. The DC:0-5 currently is the most developed standardized classification system for the assessment of mental, developmental, and relationship disorders in infancy and early childhood. The most recent edition contains significant changes that need to be evaluated for clinical applicability: the introduction of age-typical disorders, for example, represents a downward extension to identify children with early onset extreme hyperactivity and impulsivity that meet ADHD criteria, or children with early ASD symptom presentations, each of which is associated with “distress and/or functional impairment” of the child and/or his or her environment. Overall, these changes and their review are expected to improve our ability to better distinguish between transient phenomena and clinical manifestations, to shorten the diagnostic process in individual cases, to provide more rapid decision-making opportunities for more specific interventions (counseling vs. treatment), and to ultimately accelerate the allocation and release of treatment resources. Finally, it is anticipated that the results of this study can contribute to the validation of the DC:0-5. Conversely, scientific monitoring of DC:0-5 use may ultimately lead to its broader dissemination in clinics and possibly to better care for this young population in need. Finally, if relevant findings from clinical practice emerge, these can then be incorporated into future revisions of a further refined classification system.

## Figures and Tables

**Table 1:** Study protocol measurement time points and instruments. Abbreviations: ACE—Adverse childhood experiences, CBCL—Child Behavior Checklist, DC:0-5—Diagnostic Classification of Mental Health and Developmental Disorders of Infancy and Early Childhood, EBI—Parental Stress Index, EAS—Emotional Availability Scales, FraBeHa—questionnaire on treatment history, ICD-10—International Statistical Classification of Diseases and Related Health Problems, 10th Edition, Menu-Juki 0-5—questionnaire on media use, QSFS—Questionnaire of Crying Feeding Sleeping.

	Study Period		
Time Point	Enrolment	T0	T1
**Enrolment**			
Eligibility screen	all patients		
Informed consent	all patients		
**Assessments**			
Clinical interview including family and developmental history		all patients	all patients
Reports from preschool caregivers		If child is in childcare	as clinically indicated
DC:0-5 diagnoses		all patients	all patients
ICD-10 diagnoses		all patients	all patients
CBCL 1½-5		if 18 months or older	if 18 months or older
QCFS		if 17 months or younger	if 17 months or younger
EBI		all patients	
Menu-Juki 0-5		all patients	
ACE		all patients	
Frabeha			all patients
Developmental/Intelligence test		as clinically indicated	as clinically indicated
Physical exam		as clinically indicated	as clinically indicated
Motor test		as clinically indicated	as clinically indicated
Language test		as clinically indicated	as clinically indicated
EAS		as clinically indicated	as clinically indicated

## Data Availability

Not applicable.
